# Homogeneous non-selective and slice-selective parallel-transmit excitations at 7 Tesla with universal pulses: A validation study on two commercial RF coils

**DOI:** 10.1371/journal.pone.0183562

**Published:** 2017-08-21

**Authors:** Vincent Gras, Markus Boland, Alexandre Vignaud, Guillaume Ferrand, Alexis Amadon, Franck Mauconduit, Denis Le Bihan, Tony Stöcker, Nicolas Boulant

**Affiliations:** 1 CEA/DRF/Joliot/NeuroSpin/Unirs, Gif sur Yvette, France; 2 German Center for Neurodegenerative Diseases (DZNE), Bonn, Germany; 3 CEA/DRF/IRFU/SACM, Gif sur Yvette, France; 4 Siemens Healthcare, Saint Denis, France; CNRS, FRANCE

## Abstract

Parallel transmission (pTx) technology, despite its great potential to mitigate the transmit field inhomogeneity problem in magnetic resonance imaging at ultra-high field (UHF), suffers from a cumbersome calibration procedure, thereby making the approach problematic for routine use. The purpose of this work is to demonstrate on two different 7T systems respectively equipped with 8-transmit-channel RF coils from two different suppliers (Rapid-Biomed and Nova Medical), the benefit of so-called universal pulses (UP), optimized to produce uniform excitations in the brain in a population of adults and making unnecessary the calibration procedures mentioned above. Non-selective and slice-selective UPs were designed to return homogeneous excitation profiles throughout the brain simultaneously on a group of ten subjects, which then were subsequently tested on ten additional volunteers in magnetization prepared rapid gradient echo (MPRAGE) and multi-slice gradient echo (2D GRE) protocols. The results were additionally compared experimentally with the standard non-pTx circularly-polarized (CP) mode, and in simulation with subject-specific tailored excitations. For both pulse types and both coils, the UP mode returned a better signal and contrast homogeneity than the CP mode. Retrospective analysis of the flip angle (FA) suggests that the FA deviation from the nominal FA on average over a healthy adult population does not exceed 11% with the calibration-free parallel-transmit pulses whereas it goes beyond 25% with the CP mode. As a result the universal pulses designed in this work confirm their relevance in 3D and 2D protocols with commercially available equipment. Plug-and-play pTx implementations henceforth become accessible to exploit with more flexibility the potential of UHF for brain imaging.

## Introduction

Magnetic Resonance Imaging (MRI) of the brain has improved considerably in the last decade with the advent of ultra-high field (UHF) scanners (B_0_ ≥ 7T). To explore the brain non-invasively, the use of high field allows increasing the signal-to-noise ratio (SNR), reducing the acquisition times, yielding greater image resolution, but also improving the sensitivity to several effects of great importance in the study of brain organization and function. The use of high field strengths comes nevertheless with a dramatic increase in the radiofrequency (RF) field inhomogeneity which can severely degrade imaging performance. Within this context, parallel transmission (pTx) [1,2] has revealed a great potential in mitigating the transmit field inhomogeneity problem in the human head at field strengths equal or larger than 7T [3–11]. The pTx approach is the transmit analogy to parallel reception, pRx, where the MR signal is acquired with multiple receivers (phased array). Parallel reception is nowadays a standard tool in clinical MRI, e.g. for parallel imaging acceleration. While pRx increases image SNR and encodes spatial information via the reception profile, pTx provides additional degrees of freedom to shape the total excitation profile and to reduce the Specific Absorption Rate (SAR), a critical limiting measure at UHF.

Today, however, despite its power the pTx technology has remained marginally exploited. In addition to a significant financial investment in hardware to enable pTx (monitoring equipment, dedicated RF coils, etc.) and the problems engendered in patient safety management [12–14], it can be attributed mostly to the cumbersome calibration procedures (measurement of the transmit field sensitivities and pulse design) and to the expertise necessary to drive the parallel transmit coil adequately. Yet recently, a new concept of performing MRI using pTx was proposed, in which the user was spared the conventional calibration procedure [15]. In this approach, instead of computing RF pulses tailored to the subject’s actual transmit field sensitivities and static field offset map, RF pulses are designed offline, based on measured fields obtained from a group of subjects representative of a population, and blindly applied on new subjects of the considered population, without any calibration. This method, implemented experimentally with non-selective k_T_-point RF pulses [9], allowed designing so-called universal pulses (UP) whose excitation performance in terms of flip angle homogenization greatly exceeded the performance of the standard circularly polarized (CP) transmission mode commonly used in single channel transmission systems. Interestingly also, the reported performance exceeded as well the performance of the subject specific RF shim mode, although the latter technique does require the measurement of the transmit field sensitivities. The penalty in performance compared to the subject-specific tailored pulse design was found to be mild.

Although the reported results appeared promising, they were limited to non-selective excitations and involved a specific, home-made, pTx RF coil prototype, which obviously prevents from disseminating the pulses. Given the apparent necessity to provide broadband solutions to be robust against the inter-subject variability of the static field offset [15], it remained also to be determined whether the concept could be transposed to spatially selective pulses of much longer durations. The aim of the present study thus is to extend the proof of concept of UP at 7T with two commercially available pTx head coils, namely the 8TX-8RX Rapid-Biomed (RAPID Biomedical GmbH, Rimpar, Germany) and 8TX-32RX Nova (Nova medical Inc., Wilmington, MA, USA) coils, and to investigate non-selective as well as slice-selective parallel transmit pulses using larger groups of subjects than initially reported (40 versus 12 subjects). Following the approach in Ref. [15], non-selective UPs will be determined from the k_T_-point framework while slice-selective UPs will be based on multi-spoke RF pulses [5,16].

## Materials and methods

The study was conducted at two different sites (site 1 and site 2) and on two different groups of subjects. Measurements at site 1 and 2 were both made with Magnetom 7T scanners (Siemens Healthcare, Erlangen, Germany) equipped with eight-channel transmit arrays (1 kW peak power per channel). Site 1 measurements made use of the Rapid-Biomed 8TX-8RX head coil and AC84 head gradient set (50-mT/m maximum amplitude and 333-T/m/s maximum slew rate), while the measurements at site 2 were carried out with the Nova 8TX-32RX head coil and SC72 whole body gradient insert (70 mT/m maximum amplitude and 200 T/m/s maximum slew rate). At both sites, measurements were performed in the local SAR supervision mode (Siemens Tim Tx Array Step 2.3), requiring for each coil detailed numerical simulations of the electromagnetic fields on generic head models and construction of Virtual Observation Points (VOPs) [12,17] with appropriate safety margins [18]. Studies were approved by the respective local ethic committees (Comité de protection des personnes Ile-de-France de Bicêtre and Ethikkommission an der Medizinischen Fakultät der Universität Bonn) and all volunteers gave written informed consent. At each site, the respective groups consisted of 20 healthy subjects (age = 40±15 years, 10 men, and 10 women). Ten out of the 20 subjects (50% men, 50% women) were enrolled as “database subjects” and thus served the computation of UPs. The remaining ten subjects were included in the “test subject group” and their data was used to evaluate UP performance through the quantitative analysis of the simulated flip angle profiles and through examination of the images after correction of the reception profiles. “Normal” care was taken to place the subjects’ heads in the coils. It merely consisted of trying to align the space between the eyebrows with the center of the coil, although differences in neck size and table configuration did not always allow this alignment at site 1 (in this case, the two locations were put as close to each other as possible).

### MRI protocols

The acquisitions performed on the database subjects aimed at measuring the subject-specific static field offset ΔB_0_ (T) and transmit field sensitivities B_1,c_^+^ (T/V) for each transmit channel c (1 ≤ c ≤ N_c_ = 8). The ΔB_0_ distribution as well as the delineation of the brain volume were obtained by post-processing on a three-dimensional (3D) multiple gradient recalled echo (GRE) (2.5 mm isotropic resolution, matrix size 64 × 96 × 128, TR = 25 ms, 3 echoes, TE = 5, 6.5, 8 ms, TA = 3 min). The eight complex transmit field sensitivities were subsequently measured from a multi-slice interferometric turbo-FLASH acquisition (5 mm isotropic resolution, matrix size 40 × 64 × 40, TR = 20 s, TA = 4 min 40 s) [19,20]. Knowledge of the subject-based field maps on the 10 database volunteers thereby allowed designing universal pulses for each coil/site, as described in the next section.

The same field map measurements were repeated on the test subjects for retrospective control. Otherwise the MRI protocol for these subjects consisted in two different anatomical scans each repeated with two transmit modes: first in the CP mode and subsequently in the pTx mode with the universal pulses previously optimized on the database subjects (pTx-UP mode). The first scan consisted of a non-selective 3D magnetization prepared rapid gradient echo (MPRAGE) acquisition with TI = 1100 ms, TR = 2600 ms, TE = 3 ms, nominal flip angle (FA) = 5°, readout bandwidth = 260 Hz/pixel, echo train length (ETL) = 160, matrix size = 160 × 240 × 256, 1 × 1 × 1.1 mm^3^ voxels and TA = 4.5 min. The second protocol was a 40-slice 2D T_2_*–weighted GRE acquisition with TR = 1720 ms, TE = 18 ms, target FA = 30°, readout bandwidth = 40 Hz/pixel, 2 mm slice thickness, 50% slice gap, 0.5 × 0.5 mm^2^ in-plane resolution, matrix size 512 × 384. For the two acquisitions, a GRAPPA [21] acceleration factor of 2 with 24 reference lines in the phase encoding direction was used, except for the 2D GRE acquisition at site 2 which was performed with an acceleration factor of 3 (the acquisition time for the 2D GRE scans hence was decreased from 7 min at site 1 to 5 min at site 2). An additional 3D GRE CP-mode acquisition (3 mm isotropic resolution, matrix size 64 × 64 × 60, nominal FA = 5°, TR = 50 ms, TE = 2.3 ms, readout bandwidth = 300 Hz/pixel, TA = 3 min) was performed to return the reception profile of the RF coil by post-processing.

### RF pulses

For the MPRAGE acquisition in CP-mode, magnetization preparation used a hyperbolic-secant adiabatic inversion pulse of 10 ms duration with peak voltage of 140 Volts at the coil plug. The 5° (nominal FA) excitation was achieved with a rectangular hard pulse. For the 2D GRE acquisition in CP-mode, the 30° (nominal FA) excitation was performed with a standard apodized sinc pulse of time-bandwidth product 2.5. In each sequence using the CP mode, the reference voltage (V_ref_) was defined as the value required for a 0.5 ms rectangular pulse to return a FA of 90° on average in the brain isocentric axial slice, which was determined from the B_1_^+^ maps acquired on the database subjects. These measurements yielded: V_ref_ = 130 ± 5 V (mean ± std) for site 1 and V_ref_ = 95 ± 6 V for site 2.

The MPRAGE acquisition in the pTx-UP mode used a 4-ms 9 k_T_-point pulse for the inversion pulse and a 1 ms 7 k_T_-point pulse for the 5° excitation. The 2D GRE acquisitions in the pTx-UP transmission mode used 40 slice-specific 30° 5-ms long 4-spoke bipolar pulses at site 1 and 6-ms long 3-spoke monopolar pulses at site 2. At site 1, the 4-spoke pulses were designed according to the so-called “bipolar” scheme in which the spokes are played out with alternating gradient polarity. The bipolar scheme, being particularly sensitive to eddy currents, requires appropriate compensation measures for a proper implementation [6,11,22–25]. In this work, RF phase correction was used to compensate for gradient delays [26]. At site 2, preliminary tests conducted on phantom revealed that despite the employed correction scheme, the implementation of the bipolar design appeared more challenging. The bipolar scheme hence was replaced by the more robust but less efficient “monopolar” design, in which all spokes are played out with the same gradient polarity. Each spoke sub-pulse consisted of an apodized sinc-type pulse of time-bandwidth product equal to 2.5, as for the CP mode.

### Construction of the universal pulses

Universal pulses were designed to minimize the expectation value of the normalized root-mean-square error of the FA profile (FA-NRMSE) across the possible RF and static field distributions measured over the database subjects. A universal pulse **p** designed to create a uniform FA profile FA_t_ across a region of interest R (in this study, the whole brain region for non-selective pulses and the union of the 40 2D regions for the slice selective pulses) thus minimizes the quantity:

$$\epsilon (p)=\frac{1}{{\mathrm{FA}}_{t}}\langle \;{\left(\sum_{r\in R}\frac{1}{N_{V}}{\left(\mathrm{FA}\left(r\right)\text{-}{\mathrm{FA}}_{t}\right)}^{2}\right)}^{1/2}\;\rangle ,$$ (1)

where N_v_ denotes the subject-dependent number of voxels in R, FA(r) the actual FA profile for a given realization of B_1_^+^ and ΔB_0_ maps on one subject, and ⟨.⟩ the expectation value operator over the population. Assuming that the set of $B_{1}^{+}$ and ΔB_0_ measurements performed on the database subjects constitutes a representative sample of the RF and static field statistics, ϵ(**p**) can be approximated by ${(1/N}_{s})\sum_{j=1}^{N_{s}}\epsilon_{j}(p)$, where N_s_ denotes the number of subjects in the database and ϵ_j_(**p**) denotes the FA-NRMSE of pulse **p** on the j^th^ database subject. The latter expression was used as the objective function to construct the 5° non-selective, the 180° non-selective and the 30° slice selective excitations. To satisfy patient safety as well as hardware constraints, limits for the RF peak power, average power, global SAR and peak 10g SAR were defined and enforced explicitly throughout the optimization of the RF pulses [27]. From the pulse design point-of-view, the parameterization of the RF pulse **p (**k_T_-point or spoke pulses) determines the multi-dimensional variable for the optimization. With N_p_ denoting the number of sub-pulses and n_sl_ the number of slices (1 and 40 for the non-selective and selective pulses respectively), **p** is composed of i) n_sl_ × N_p_ × N_c_ complex RF coefficients and ii) N_p_ × 3 (non-selective case) or n_sl_ × N_p_ × 2 (selective case) real coefficients for the sparse k-space locations. In this work, all coefficients (complex sub-pulse coefficients and transmit k-space locations) were optimized jointly under SAR and power constraints using the active-set algorithm [28,29]. Pulse design was conducted by using the small tip angle approximation for the 5° non-selective and 30° slice-selective pulses while numerical integration of the Bloch equation were conducted for the inversion pulse. To reduce computation times, the latter operation was ported on a Nvidia (Nvidia, Santa Clara, CA, USA) Tesla K40 graphics processing units card. Ultimately, final pulse performance was always verified by using numerical integrations of Bloch equation.

### Flip angle simulations

The subject-specific $B_{1}^{+}$ and ΔB_0_ measurements performed on the test subjects fed Bloch equation simulations to yield FA profiles for the CP and universal pulses integrated in the MPRAGE and 2D GRE sequences. For comparison, additionally, subject-specific static RF-shim and tailored (k_T_-point or spoke) pulses were computed by minimization of the FA-NRMSE under the same RF power and SAR constraints as for the design of the pTx-UPs. For each pulse (5° non-selective, 180° non-selective, 30° selective), the CP, pTx-UP, RF-shim modes and subject tailored pTx spoke or k_T_-point pulses (obtained through the minimization of the subject-specific FA-NRMSE) were compared in terms of their respective FA-NRMSE performance.

## Results

### MPRAGE and 2D GRE comparisons

**Fig 1:**
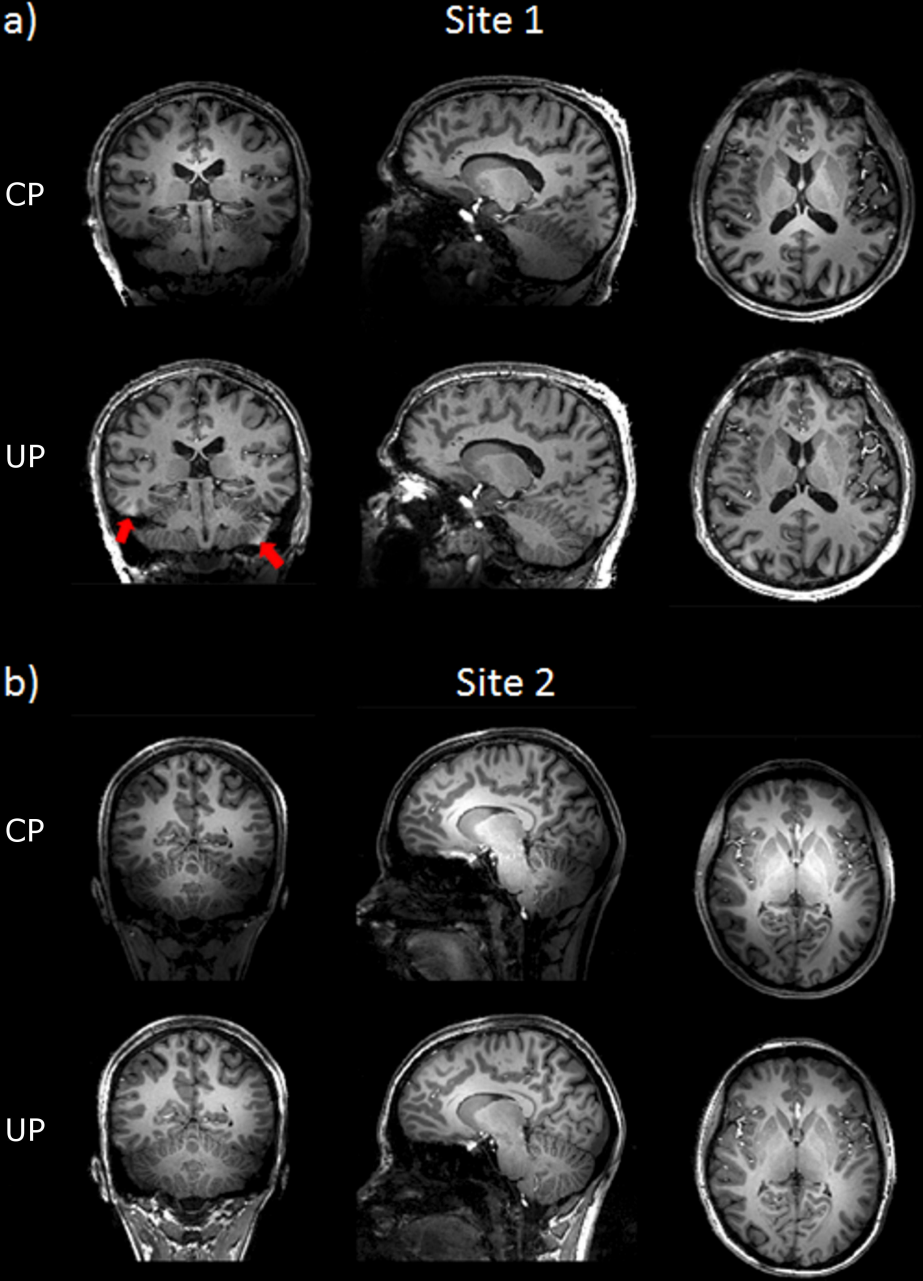
Circularly-polarized versus pTx universal pulse comparison in two MPRAGE datasets acquired at site 1 and site 2 respectively. MPRAGE image comparison in sagittal, coronal and axial planes for a) a test subject of site 1 (Rapid-Biomed coil) and b) a test subject of site 2 (Nova coil). Especially at site 2, the bright center removal by UPs is more pronounced. Moreover at the same site, one can observe a larger coverage in the neck area due to the increased broadband behavior of the UPs. In both cases, the signal and contrast homogeneity is dramatically improved in the cerebellum, temporal lobes and top of the brain when using UPs. The utilization of UPs at site 1 may cause however the occasional emergence of small susceptibility artifacts in some areas (red arrows), e.g. at the interface with air cavities at the basis of the skull. The coronal image of the subject scanned at site 2 displays a residual signal heterogeneity on both sides of the cerebellum suggesting some imperfection in excitation pattern of the 5° UP.

**Fig 2:**
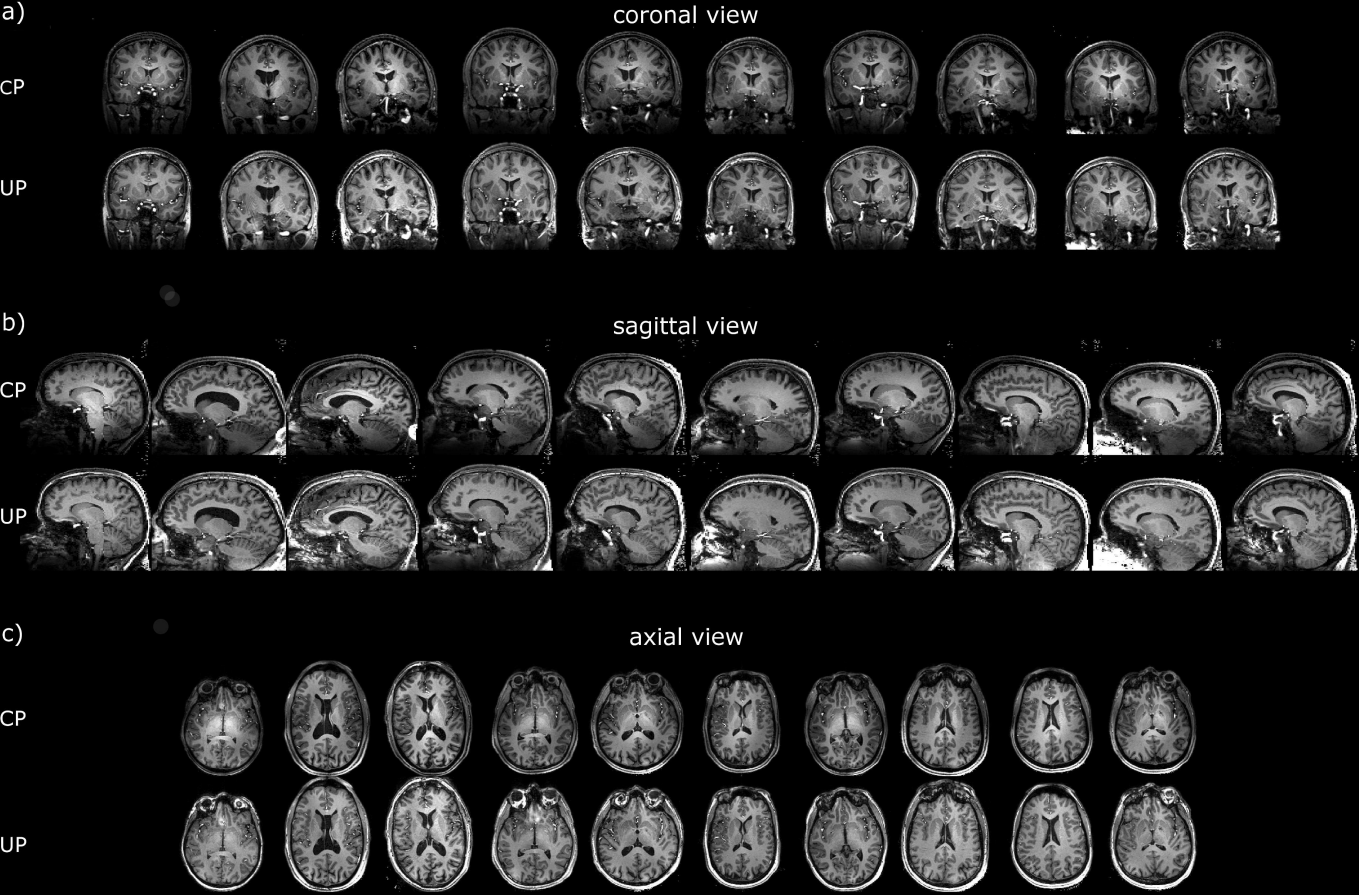
Receive profile corrected MPRAGE scans on the test subjects at site 1. Coronal (a), sagittal (b) and axial (c) views of the MPRAGE scans (receive profile corrected) obtained on the test subjects at site 1 (Rapid-Biomed coil) with the CP and pTx-UP mode. In general, for the entire test group, the overall signal and contrast homogeneity is clearly improved with the use of UPs. The bright center clearly visible in the CP-mode is well reduced with the proposed excitation mode. In most of the test subjects, a clear improvement of the contrast between white matter and gray matter is visible in the cerebellum and the left and right temporal lobes. The coronal views of subjects 3 and 8 and the sagittal views of subjects 1, 3, 7 and 8 display susceptibility (i.e., ΔB_0_-induced) artefacts. These are present with the pTx-UP but not with the CP mode. The sagittal views of subjects 2, 8 and 9 show signal hyper-intensities and contrast loss in the lower part of the cerebellum, symptomatic of an incomplete inversion of the magnetization. The problem is apparent for the subjects whose head position was low in z and is linked with the rapid decrease of the coil transmit efficiency in z.

**Fig 3:**
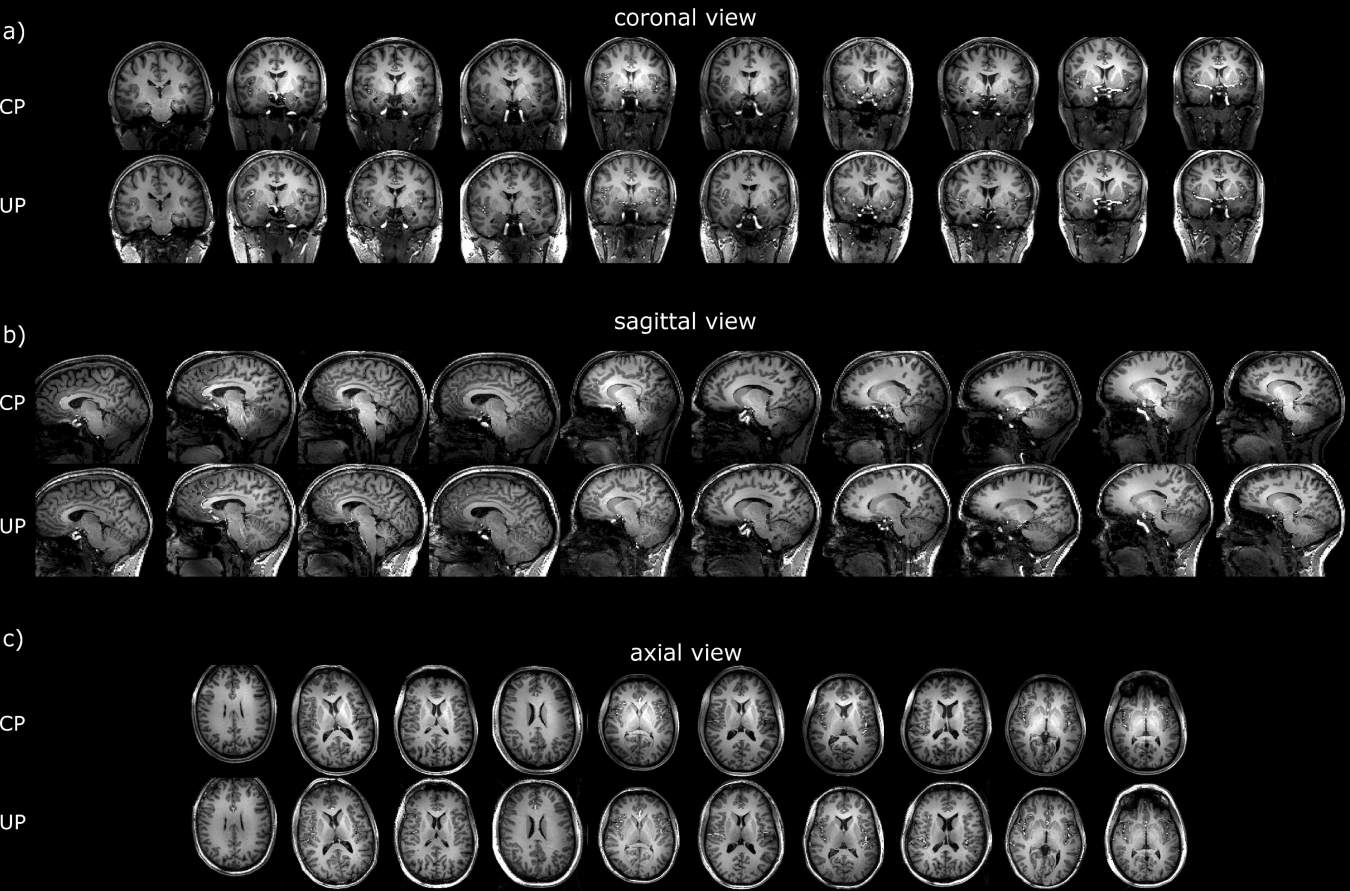
Receive profile corrected MPRAGE scans on the test subjects at site 2. Coronal (a), sagittal (b) and axial (c) views of the MPRAGE scans (receive profile corrected) obtained on the test subjects at site 2 (Nova coil) with the CP- and the pTx-UP modes. Compared to site 1, the bright center of the CP mode appears slightly more pronounced, but the CP contrast (mostly driven by the inversion efficiency) is generally better preserved in the lower part of the brain. Yet, similar observations as for site 1 can be made regarding the signal and contrast homogeneity improvements with the utilization of UPs. In contrast with the MPRAGE images acquired at site 1, the susceptibility-induced artefacts pointed out in Fig 2 is much more reduced at site 2, making the images virtually artefact-free. As suggested in the discussion, this improvement is enabled by the higher transmit efficiency of the coil used as site 2.

**Fig 4:**
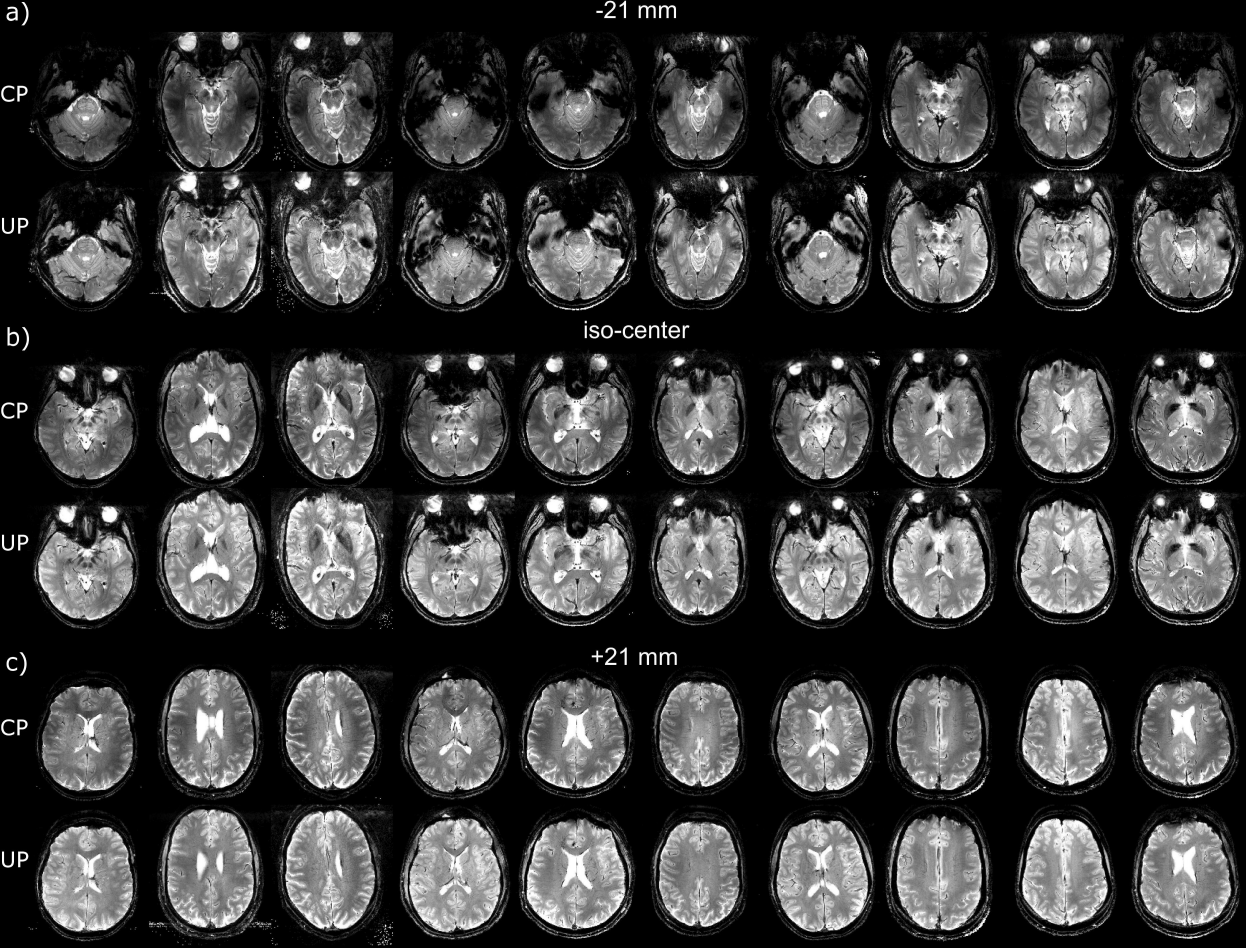
Receive profile corrected 2D GRE scans on the test subjects at site 1. Comparison of the 2D GRE acquisitions (receive profile corrected) performed at site 1 (Rapid-Biomed coil) in the CP and pTx-UP (4-spoke bipolar pulse) modes. a) -21 mm, b) iso-center and c) +21 mm out of the 40 acquired slices are shown. As previously mentioned, the transmit efficiency of the CP mode decaying rapidly in the lower part of the brain, the signal enhancement is important in the lowest slices. Still, in b) and c), the slight transmit fall-off in the left-right directions in the CP mode appear well corrected with the pTx UPs.

**Fig 5:**
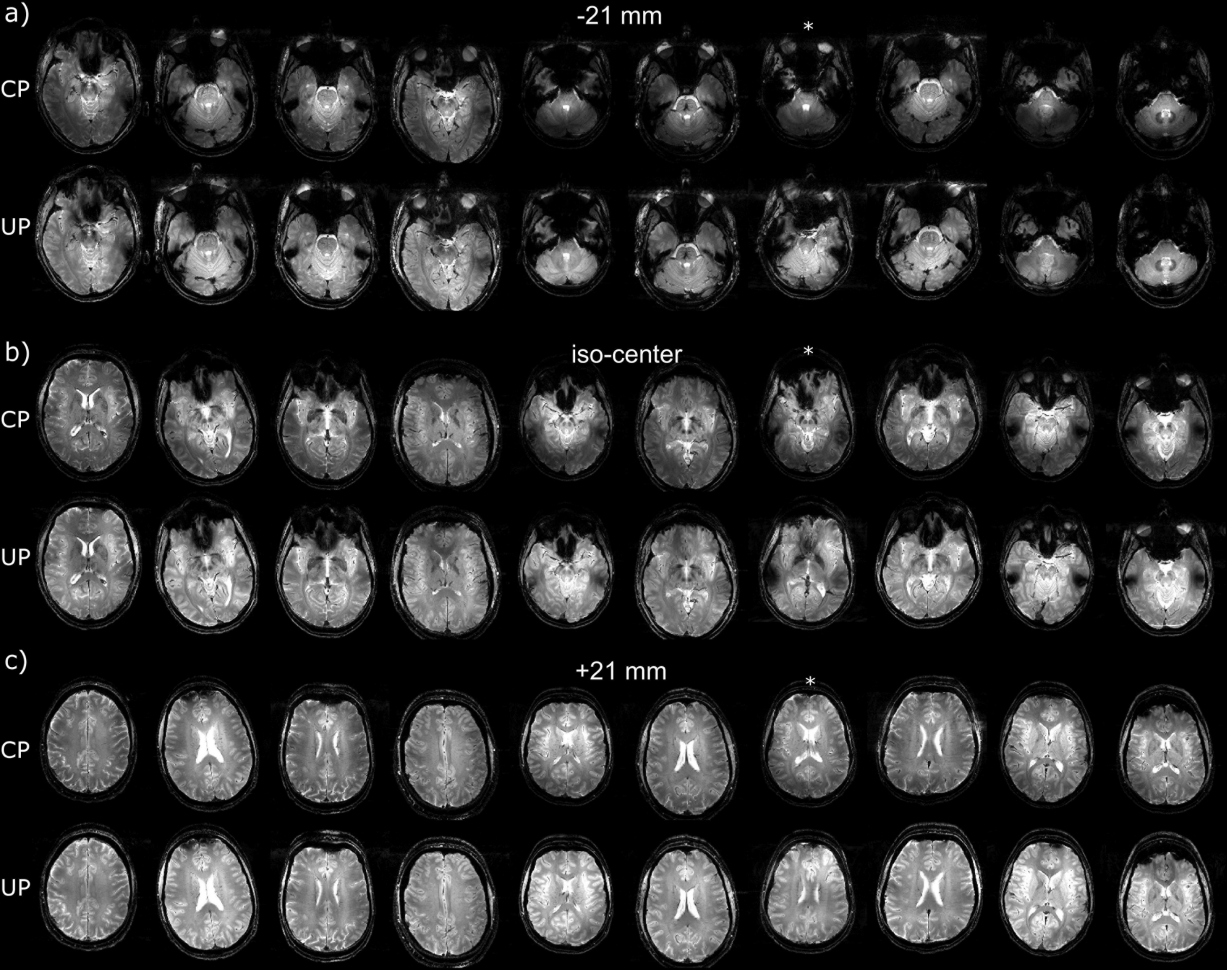
Receive profile corrected 2D GRE scans on the test subjects at site 2. Comparison of the 2D GRE acquisitions (receive profile corrected) performed at site 2 (Nova coil) with the CP and pTx-UP (3-spoke monopolar pulse) modes. a) -21 mm, b) iso-center and c) +21 mm out of the 40 acquired slices are shown. As for site 1, the gain brought by the utilization of UPs is mostly seen in the lower slices where the transmit efficiency of the CP mode drops rapidly as well. For subject 7 (marked with an asterix), due to an interruption of the exam and the renewed placement of the subject in the scanner between the acquisitions in the CP- and the pTx-UP mode, the position consistency was compromised.

For two test subjects scanned respectively at sites 1 and 2, differences between the MPRAGE in CP- and the pTx-UP modes are highlighted in selected orthogonal views of the brain in Fig 1. The results for all 10 test subjects for the same scan are also provided for both coils in Figs 2 and 3 respectively. Similar comparisons are provided for the 2D GRE protocol in Figs 4 and 5. All images shown are corrected for the reception profile. Hence, up to the precision of the receive profile correction procedure, the signal inhomogeneity displayed in Figs 1–5 is representative of the transmission inhomogeneity only. Incidentally, the same figures reveal to some extent the variability in head geometry, size and position. For the MPRAGE sequence, except for Fig 1, the same (scanner coordinates) coronal, sagittal and axial slices are shown for all test subjects. For the 2D GRE sequence, 3 slice positions (same scanner coordinates) located at the bottom, middle and top of the brain were selected and displayed again for all test subjects.

### Retrospective control with flip angle simulations

**Fig 6:**
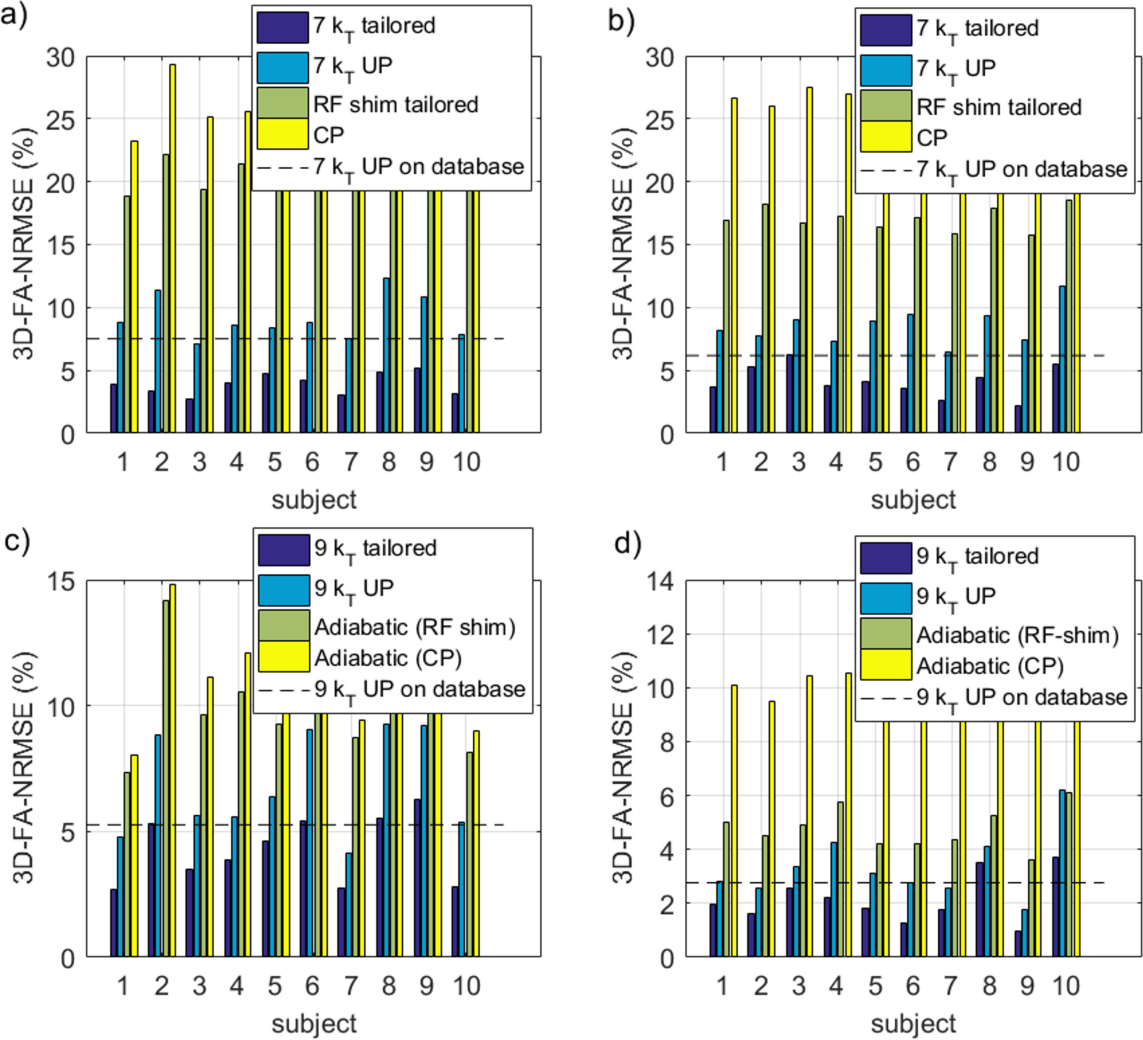
Pulse performance comparisons for the non-selective 5° and 180° pulses. FA-NRMSE simulations of a,b) the non-selective 5° CP, 7 k_T_-point universal, subject specific RF-shim and subject specific 7 k_T_-point pulses and c,d) non-selective 180° adiabatic CP, adiabatic RF-shim, 9 k_T_-point universal and subject specific 9 k_T_-point pulses designed at site 1 (a,c) and site 2 (b,d). The dashed line represents the average UP 3D-FA-NRMSE on the database subjects. The UP NRMSE at both sites on all test subjects is noticeably lower than the 13% threshold corresponding to the CP mode at 3T with a volume coil [30].

**Fig 7:**
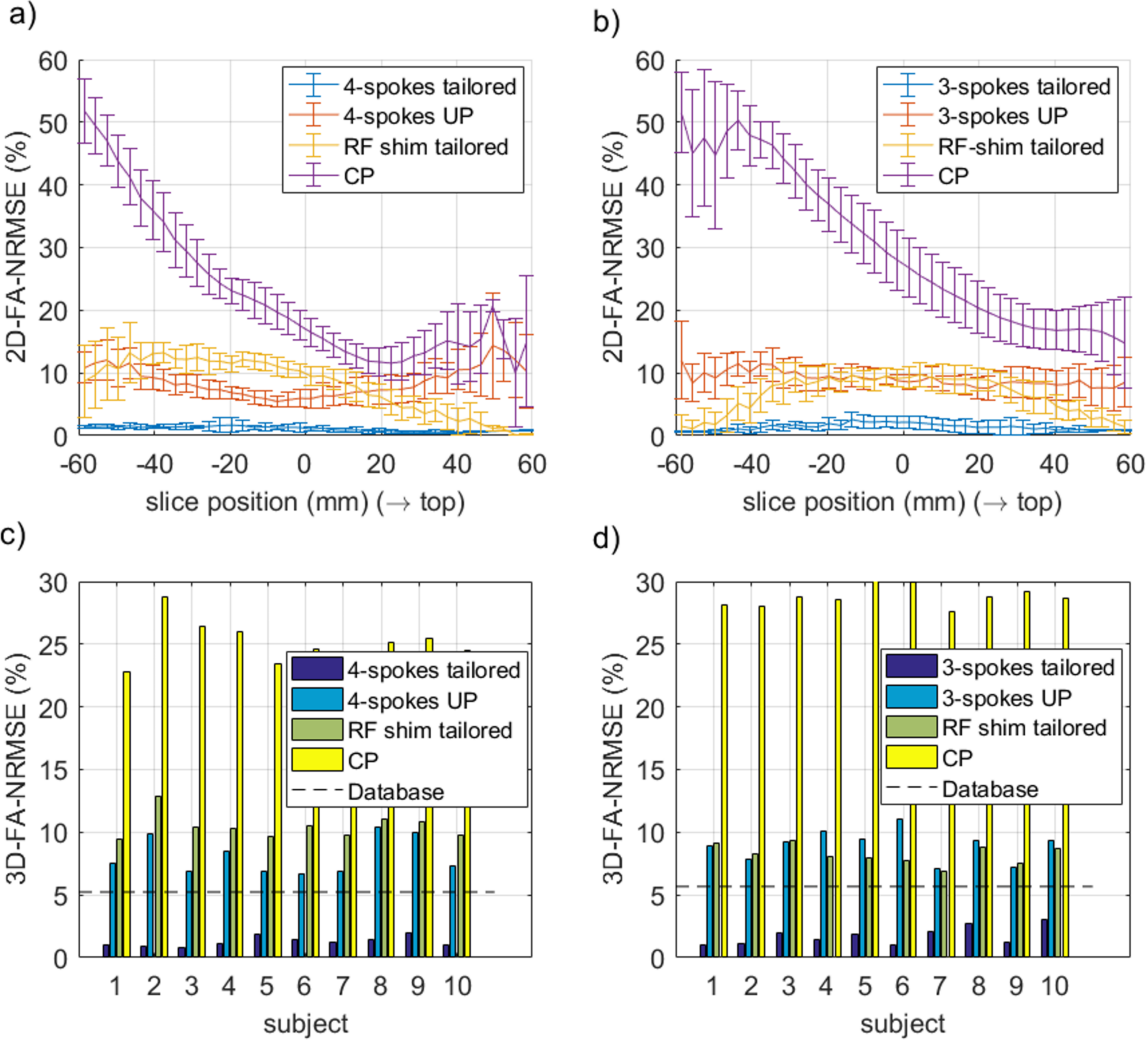
Pulse performance comparisons for the slice-selective 30° pulses. FA-2D-NRMSE (a-b) and 3D-FA-NRMSE (c-d) simulations of the 30° slice selective RF pulses designed at site 1 (a,c) and site 2 (b,d) and for all test-subjects. The CP and the 3- or 4-spoke pTx-UP were the excitation modes tested experimentally. For comparison, the subject-specific, slice-specific optimized RF-shim and 3- or 4-spoke designs were included as well. The dashed line represents the average 3D-FA-NRMSE obtained over the database subjects with the UPs. For both coils, the spoke UPs perform systematically better than (site 1), or comparably to (site 2) the subject-specific 2D RF shims. Also the 3D-FA-NRMSE is always smaller than the 13% threshold, corresponding to the CP mode inhomogeneity at 3T in the brain with a volume coil [30].

The FA-NRMSE of the 5° pulses (CP, UP, subject specific RF-shim and subject-specific optimized k_T_-point) is reported in Fig 6A and 6B for sites 1 and 2 respectively. In Fig 6C and 6D, the same comparison is provided for the non-selective 180° pulses. For the slice-selective 30° RF pulses, the slice-by-slice FA-NRMSE (2D-FA-NRMSE) is reported in Fig 7A and 7B. There, for each slice, the average FA-NRMSE and its standard deviation across the 10 test subjects (error bar) is reported for the CP, universal, subject-specific RF-shim, and subject-specific optimized multi-spoke pulses. In the bar-plots of Fig 7C and 7D, the global FA-NRMSE obtained by pooling all slices together (3D-NRMSE) is reported for each test subject, and again for both coils and sites. It can be noted that the CP-mode whole brain FA-NRMSE typically exceeded 25% whereas it remained below 10% in the inversion case and below 13% in the small FA case with UPs. That quantitative result, is supported by the image comparisons provided in Figs 1–5 which display a clear systematic improvement of the excitation uniformity with pTx-UP compared to the CP transmission mode and additionally inform on the spatial distribution of the residual excitation errors of both modes of transmission. Interestingly also, it appears that the 180° pTx-UP returned better whole brain FA-NRMSEs than the adiabatic hyperbolic secant pulses (see Fig 6C and 6D). The MPRAGE image comparisons provided in Figs 1–3 again support this result. Indeed, in a large part of the cerebellum and occasionally in temporal regions, the contrast between white matter and gray matter is lost with the CP excitation. Although imperfections occasionally remain at site 1 on the lowest part of the cerebellum, the inversion efficiency is significantly improved with the utilization of the 180° pTx-UP, as shown by the contrast improvement in the aforementioned regions.

The average 3D-FA-NRMSE values (mean ± std) obtained for the 5°, 180° and slice-selective 30° pulses on the test subjects is summarized in Table 1 (full 3D-FA-NRMSE statistics are also available in S1 Table). Those were further exploited to attempt giving the 99% confidence interval (see Table 2) on the average performance of the pTx-UP mode over a much larger population for the two coils investigated. It can be seen in particular that the upper bound of the 99% confidence interval does not exceed 11% FA-NRMSE.

**Table 1:** Average 3D-FA-NRMSE values of the 5°, 180° and slice-selective 30° pulses.

	Site 1			Site 2		
	5° ns	180° ns	30° ss	5° ns	180° ns	30° ss
**CP**	26.1±2.4	12.1±2.6	25.1±1.7	27.4±1.0	10.5±0.8	28.8±0.9
**ST RF-shim**	21±1.6	10.3±1.9	10.5±1.0	17.1±0.9	4.8±0.8	8.3±0.8
**UP**	9.2±1.7	6.8±2.0	8.1±1.5	8.6±1.5	3.4±1.2	8.3±1.2
**ST k**_**T**_**/spokes**	3.8±0.8	4.3±1.3	1.3±0.4	4.1±1.3	2.1±0.9	3.6±1.4

Average and standard deviation of the 3D-FA-NRMSE (in %) values obtained at sites 1 and 2 over 10 test subjects for the subject-tailored (ST) RF-shim-, universal- and subject-specific k_T_-point or spoke pulses implementing the non-selective (ns) 5° and 180° and the slice-selective (ss) 30° excitations. For the 180° pulse, the CP and ST RF-shim modes are played with a 10 ms hyperbolic secant (HS) shape.

**Table 2:** Confidence interval of the 3D-FA-NRMSE for the 5°, 180° and slice-selective 30° pTx universal pulses.

	Site 1	Site 2
**5° ns**	9.2±1.8	8.6±1.5
**180° ns**	6.8±2.1	3.4±1.3
**30° ss**	8.1±1.5	8.3±1.2

99% confidence interval for the average whole brain 3D-FA-NRMSE of the pTx-UP transmission mode over the entire healthy adult population for both sites/coils. The confidence interval is given by $\bar{x}\pm t_{0.01}\frac{\sigma_{x}}{\sqrt{n}}$, where n = 10 is the sample size (the number of test subjects), $\bar{x}$ and σ_x_ are respectively the mean value and standard deviation of the FA-NRMSE over the test subjects, and where t_0.01_ = 3.23 satisfies Prob(|T| > t_0.01_) = 0.01 for the random variable T whose statistics is the Student’s t-distribution with n − 1 degrees of freedom.

## Discussion

The FA-NRMSE comparisons summarized in Table 1 show that the proposed UP approach clearly outperforms the CP and RF shim modes in 3D while for the slice-selective 30° pulses, the whole brain performance (3D-FA-NRMSE) of UPs is at least comparable to the subject and slice specific RF-shims. The FA-NRMSE confidence intervals given in Table 2 furthermore indicate that the UPs designed in this study returned *on average* in a healthy adult population (age 40±15 years) whole-brain FA-NRMSEs below 11% with 99% confidence (the worst case being the 5° non selective pulse for site 1). Comparing this number with the inhomogeneity of the CP-mode B_1_^+^ field within the brain at 3T [30], namely 13%, the proposed pTx-UP mode allows to recover a uniformity of excitation better than that of a volume coil at 3T, yet without a tedious calibration procedure. Especially for the slice-selective pulses, a penalty in performance with the UPs compared to subject-specific more sophisticated approaches yet naturally remains. The inhomogeneity with the UPs, however, remained for both non-selective and selective pulses below the 13% threshold of a volume coil at 3T in the human brain [30]. At this point, it is worth noting however that a same FA-NRMSE value can lead to many different FA distributions (including worst case scenarios) because NRMSEs provide only a unique quality number. It follows that a more qualitative analysis of the images is very useful to grasp the full implication of the utilization of UPs in the perspective of clinical practice. For further optimality, partitioning a bigger database according to relevant criteria, such as for instance head size or position in the z direction, with corresponding pulses could increase the performance of the UPs [31]. At site 1, constraints due to the table configuration and differences in neck size indeed prevented from positioning the subjects identically, which could hence lead to better results with database segmentations.

Direct flip angle measurements in this study were not performed in vivo due to incompressible exam durations. Such types of measurements, however, have been reported in many other works in both small and large flip angle regimes and across several groups, field strengths and vendors [9,28,32–35], thus confirming proper hardware implementation with state of the art equipment. The examination of the receive profile-corrected MPRAGE and 2D GRE images demonstrated a very good recovery of the signal and constrast homogeneity across the whole brain but allowed identifying some specific imperfection. The susceptibility (i.e., ΔB_0_-induced) artefact pointed out in the MPRAGE images acquired at site 1 (see Fig 2) was one of them. Yet a positive aspect and an incidental consequence of the broadband behavior of the UPs [15] is a larger coverage, yielding greater signal in the neck area (Figs 1 and 3), thereby making structures in this region visible (e.g. muscles, tendons).

The susceptibility-induced artefact mentioned above, clearly visible in the MPRAGE images acquired at site 1 while absent at site 2, most likely arises from the lower transmit efficiency of the RF coil used at site 1(V_ref_ = 130 V at site 1 vs 95 V at site 2). As a result, for the same peak RF amplitude of 160 V, a comparatively higher contribution of the ΔB_0_ term in the spin dynamics is observed at site 1 than at site 2. In other words, the transmit efficient is an important requirement to enable broadband solutions necessary to tackle inter-subject B_1_ and ΔB_0_ map variability [15]. This raises the importance of good static field homogenization and coil efficiency for application of UPs. The problem on the other hand could be addressed from the pulse design perspective, where a tradeoff between spatial and spectral uniformity of the UP could be potentially enforced in the objective function (Eq 1).

Although the construction of the proposed pTx universal pulses was mostly driven by radio-frequency considerations, the RF coil properties, the gradient slew-rate limits and the eddy-current characteristics of the system also came into play for the slice-selective case. At site 2, the implementation of the bipolar design appeared difficult. A possible explanation for this is that the coil used at site 2 carries more eddy currents on its conductors and RF shield than the one equipping site 1. The more robust monopolar design hence was used at site 2. Due to the time penalty engendered by the fly-back trajectory, the number of spoke sub-pulses in this case was reduced to 3 (versus 4 at site 1), partly explaining the loss of performance in simulation when comparing the two sites.

For the universal pulse design, we chose in our objective function to minimize the average (NRMSE) performance over the database subjects. Worst-case NRMSE optimization could be performed likewise [15]. Although the latter appears appealing conceptually, we found that a worst-case approach could, perhaps unsurprisingly, penalize the average performance non-negligibly and even not perform the desired task on the test subjects. Table 3 indeed reports the simulated NRMSEs over the test and database subjects for the two optimization strategies, i.e. minimization of the average and worst-case NRMSEs respectively, and for the two different coils. The NRMSEs over the database subjects perform as expected, i.e. according to the cost-function used in the optimization. On the other hand, at site 2, minimizing the worst-case NRMSE over the database subjects does not guarantee a lower worst-case over the test subjects than the one obtained with the other optimization method. This illustrates the greater sensitivity of the results to subject-specific details in the RF field and static field offset maps when worst-case metrics are employed. Yet, e.g. with RF field and static field offset segmentation strategies [31], we do not rule out the possibility of using other optimization metrics to target more specifically radiologists’ needs.

**Table 3:** Comparison of the original and the proposed universal pulse optimization metrics.

	UP construction method	<NRMSE> (%)	Max(NRMSE) (%)
**Site 1**	min(<NRMSE>)	9.6 (7.7)	14.2 (9.9)
	min(max(NRMSE))	10.6 (8.8)	13.1 (9.1)
**Site 2**	min(<NRMSE>)	8.6 (6.2)	**11.7 (8.1)**
	min(max(NRMSE))	9.6 (7.5)	**12.4 (7.6)**

NRMSE results over the test subjects of the two optimization metrics and for the two different coils. The second column indicates the optimization strategy (minimization of the average NRMSE (Eq 1) versus worst-case NRMSE over the database subjects (Eq 3 in ref. [15])). Results for the database subjects are indicated between parentheses. For site 2, the rightmost column (numbers in bold) illustrates the sensitivity of the results to subject-specific details in the RF transmit field and static field offset maps. In this case, the minimization of the worst-case NRMSE over the database subjects indeed does not guarantee a better worst-case NRMSE than the proposed design (minimization of the average NRMSE, see Eq 1) on the test-subjects.

For the CP-mode, the reception profile of the phased array is known to partly compensate for the transmit inhomogeneity [36]. Hence from a global signal homogeneity perspective, it is not necessarily desirable to apply any receive profile correction in this transmit configuration. In contrast with this, since universal pulses are designed to produce homogeneous excitation patterns, correction of the receive profile in this case is perfectly adequate. As a result, the image comparisons provided in Figs 1–5 thus may penalize the CP mode performance in favor of the UPs. Yet, from the SNR point-of-view the proposed comparison illustrates very objectively the gain that is brought by UPs. Moreover, the "accidental" cancelation of the CP-mode transmit inhomogeneity with the reception profile of the head coil array cannot compensate for spatially varying contrast and thus does not guarantee the optimality of the contrast-to-noise ratio.

## Conclusion

In this work, we have designed non-selective and slice-selective parallel-transmit universal pulses for two commercially available head coils. The proposed RF pulses were able to mitigate the RF inhomogeneity problem in the human brain at 7T without prior calibration of the subject-specific transmit RF fields. Our pulse performance analysis, based on two groups of 10 test subjects (one group per site) indicates with 99% confidence that the FA-NRMSE of the designed UPs does not exceed 11% on average in healthy adults (age 40±15 years) for non-selective and slice-selective pulses. The coil-specific UPs, tested at both sites, on all test subjects in standard MPRAGE and 2D-GRE protocols, demonstrated a clear image quality improvement in terms of flip angle and contrast uniformity in comparison with the CP-mode, confirming the robustness of the approach and the possibility of using and distributing such calibration-free solutions for clinical routine applications.

## Supporting information

S1 Fig Non-selective and selective UP representations. Left column: site 1 (Rapid-Biomed); right column: site 2 (Nova Medical): a-b) 5° non-selective 7 k_T_-points, c-d) 180° non-selective 9 k_T_-points, e-f) 30° multi-spoke pulses. Both RF amplitude and x, y and z gradient amplitudes are shown.
(TIF)

S2 Fig Database of transmit RF field maps at site 1. Magnitude (left image) and phase (right image) of the transmit RF field maps (one column of image per subject and one row of image per transmit channel) of the database subject at site 1 (Rapid-Biomed).
(PNG)

S3 Fig Database of transmit RF field maps at site 2. Magnitude (left image) and phase (right image) of the transmit RF field maps (one column of image per subject and one row of image per transmit channel) of the database subject at site 2 (Nova Medical).
(PNG)

S1 Table 3D-FA-NRMSE statistics. 3D-FA-NRMSE values of the non-selective 5°, non-selective 180° and selective 30° pulses reported separately across all test subjects and for the two sites.
(XLS)
