# Supplementary figures and images for: Homogeneous non-selective and slice-selective parallel-transmit excitations at 7 Tesla with universal pulses: A validation study on two commercial RF coils
Source: PLoS One. 2017 Aug 21;12(8):e0183562. doi: 10.1371/journal.pone.0183562 (PMC5565195; doi:10.1371/journal.pone.0183562)

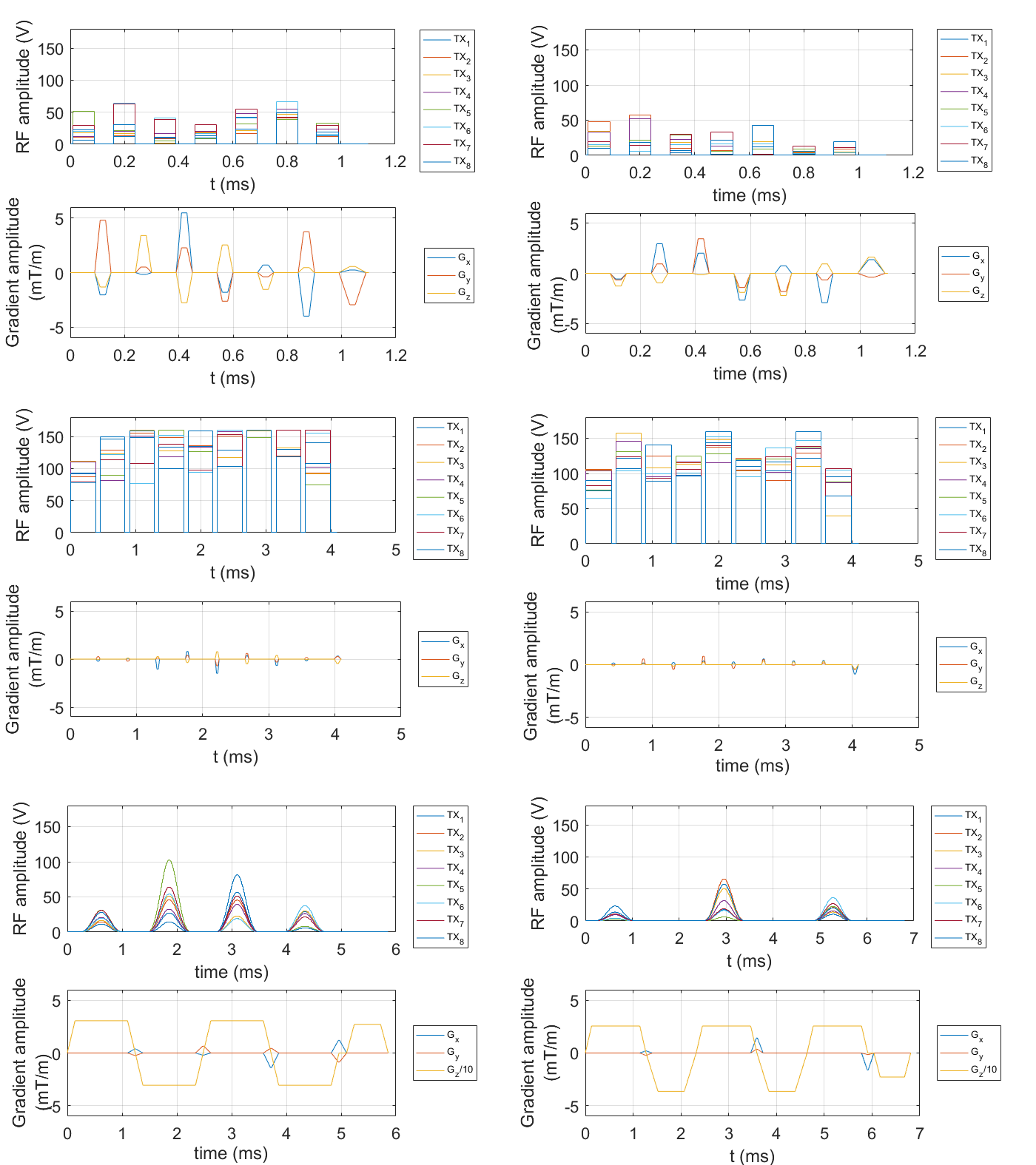

Supplement: S1 Fig — Left column: site 1 (Rapid-Biomed); right column: site 2 (Nova Medical): a-b) 5° non-selective 7 kT-points, c-d) 180° non-selective 9 kT-points, e-f) 30° multi-spoke pulses. Both RF amplitude and x, y and z gradient amplitudes are shown. (TIF) [file pone.0183562.s001.tif]

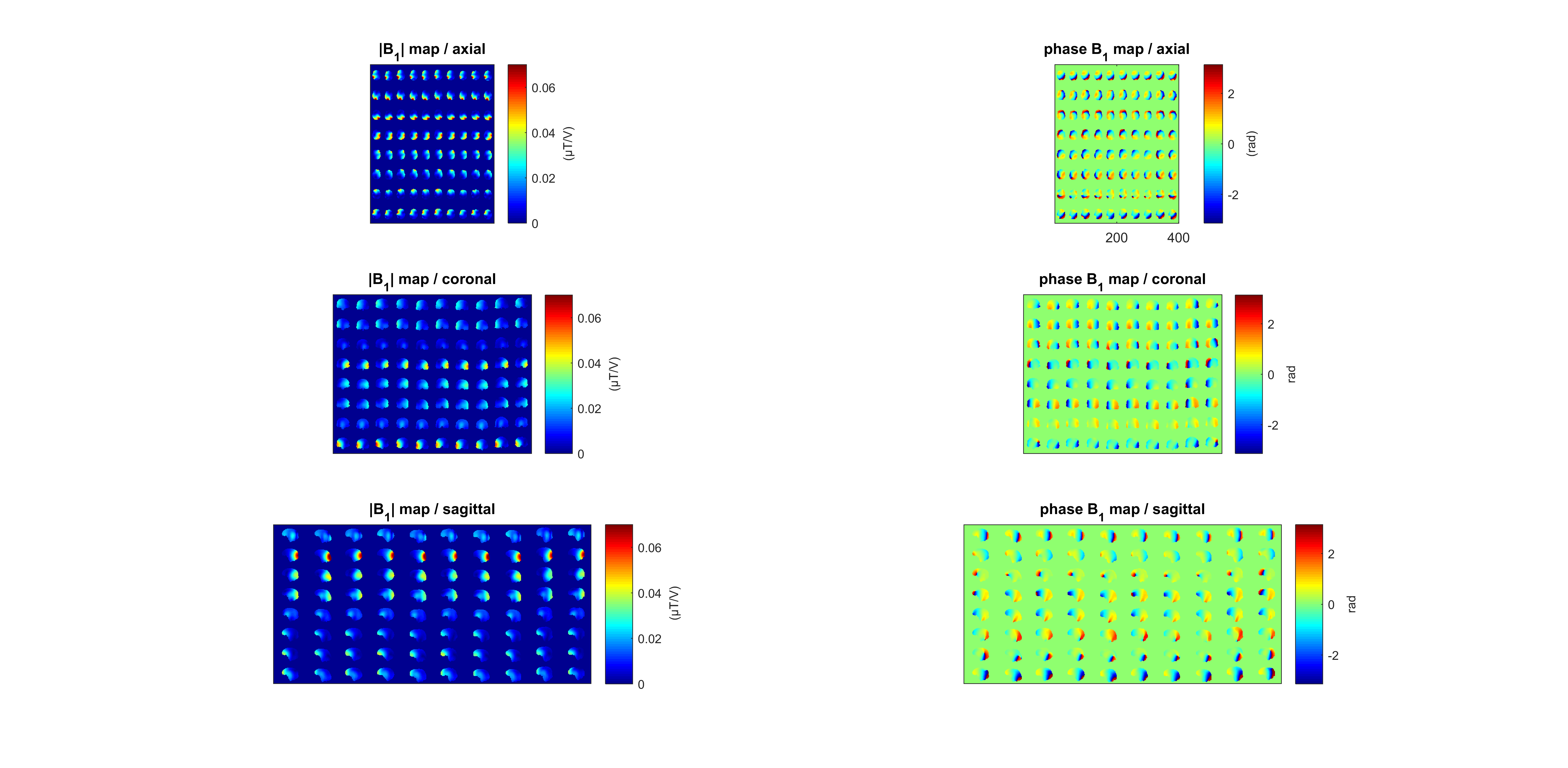

Supplement: S2 Fig — Magnitude (left image) and phase (right image) of the transmit RF field maps (one column of image per subject and one row of image per transmit channel) of the database subject at site 1 (Rapid-Biomed). (PNG) [file pone.0183562.s002.png]

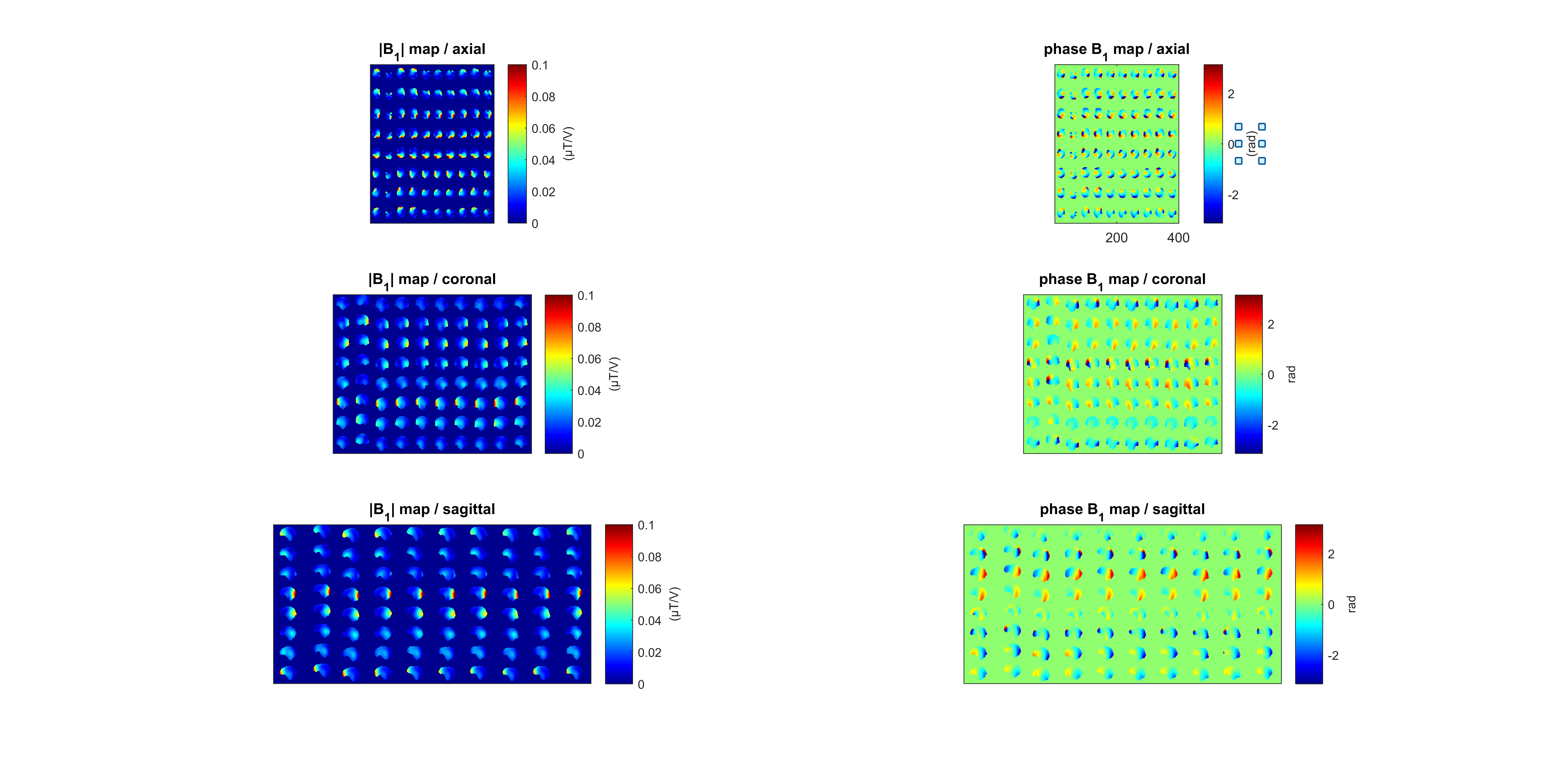

Supplement: S3 Fig — Magnitude (left image) and phase (right image) of the transmit RF field maps (one column of image per subject and one row of image per transmit channel) of the database subject at site 2 (Nova Medical). (PNG) [file pone.0183562.s003.png]
